# Aberrant Anaplastic Lymphoma Kinase Activity Induces a p53 and Rb-Dependent Senescence-Like Arrest in the Absence of Detectable p53 Stabilization

**DOI:** 10.1371/journal.pone.0017854

**Published:** 2011-03-14

**Authors:** Fiona Kate Elizabeth McDuff, Suzanne Dawn Turner

**Affiliations:** Division of Molecular Histopathology, Department of Pathology, University of Cambridge, Cambridge, Cambridgeshire, United Kingdom; University of Barcelona, Spain

## Abstract

Anaplastic Lymphoma Kinase (ALK) is a receptor tyrosine kinase aberrantly expressed in a variety of tumor types, most notably in Anaplastic Large Cell Lymphoma (ALCL) where a chromosomal translocation generates the oncogenic fusion protein, Nucleophosmin-ALK (NPM-ALK). Whilst much is known regarding the mechanism of transformation by NPM-ALK, the existence of cellular defence pathways to prevent this pathological process has not been investigated. Employing the highly tractable primary murine embryonic fibroblast (MEF) system we show that cellular transformation is not an inevitable consequence of NPM-ALK activity but is combated by p53 and Rb. Activation of p53 and/or Rb by NPM-ALK triggers a potent proliferative block with features reminiscent of senescence. While loss of p53 alone is sufficient to circumvent NPM-ALK-induced senescence and permit cellular transformation, sole loss of Rb permits continued proliferation but not transformation due to p53-imposed restraints. Furthermore, NPM-ALK attenuates p53 activity in an Rb and MDM2 dependent manner but this activity is not sufficient to bypass senescence. These data indicate that senescence may constitute an effective barrier to ALK-induced malignancies that ultimately must be overcome for tumor development.

## Introduction

Anaplastic Lymphoma Kinase (ALK) is a member of the insulin receptor tyrosine kinase family, whose expression is predominately limited to the developing nervous system and at low levels in adult neuronal cells [1], [2], [3]. Whilst little is known of the physiological function of ALK, it is rapidly gaining recognition as an important oncogene in a diverse range of tumor types, including Anaplastic Large Cell Lymphoma (ALCL), melanoma, breast cancer, non-small cell lung carcinoma, neuroblastoma, inflammatory myofibroblastic tumors and esophageal squamous cell carcinoma although its involvement in some of these cancers is still somewhat controversial [1], [4], [5], [6], [7], [8], [9], [10], [11], [12], [13], [14], [15], [16]. Deregulated ALK expression in each of these cases is the consequence of either activating point mutations of the full-length protein or chromosomal aberrations including translocations and inversions which juxtapose the kinase region of ALK to an oligomerization domain encoded in the partner gene. The oncogenic potential of ALK has been best demonstrated in ALCL in which it is expressed as a fusion protein, predominantly juxtaposed to NPM [1]. The NPM portion of NPM-ALK provides an oligomerization interface to permit dimerization of NPM-ALK monomers, followed by autophosphorylation and constitutive activation [17]. A direct role for NPM-ALK in cellular transformation has been shown both *in vitro and in vivo*, and such studies have shed light on the mechanism of malignant transformation by this oncoprotein [18], [19]. These mechanisms include activation of several downstream signal-transduction pathways, which regulate cell survival, proliferation and migration [18], [20], [21].

The t(2;5) encoding NPM-ALK and the corresponding transcripts have been detected in the peripheral blood of apparently healthy individuals at a frequency of 48–65% [22], [23]. These findings are in line with the presence of other known oncogenic translocations in tumor-free donors, such as BCR-ABL [24], [25], IgH-Bcl2 [26], [27] and PML-RARα [28]. These data indicate that the presence of the oncogenic translocation *per se* is not sufficient for tumorigenesis; rather, additional molecular events are likely required for the cell to become fully transformed and to bypass barriers such as apoptosis or oncogene-induced senescence (OIS) [29]. Further evidence for multi-step NPM-ALK-induced lymphomagenesis stems from studies of NPM-ALK-transgenic mice in which the latency to disease in some cases can be anything from 9 months to 2 years suggesting a requirement for cooperative mutations [30], [31]. Hence, these findings imply that NPM-ALK is necessary but not sufficient for lymphoma development.

The p53 and Rb tumor-suppressor genes are two of the most frequently mutated genes in human cancer, and the proteins encoded by these genes have multiple tumor-suppressor functions, not least their roles in apoptosis, transient cell-cycle arrest and senescence [32]. In order to investigate whether aberrant ALK activity has the potential to trigger cellular mechanisms that prevent oncogenic transformation, NPM-ALK was expressed in primary murine embryonic fibroblasts (MEFs), a genetically amenable and well-characterized system that has been used extensively in the study of oncogene-induced senescence and apoptosis. Particular attention was paid to the potential roles of the p53 and Rb pathways in limiting NPM-ALK-induced transformation.

We show that NPM-ALK induces a cell-cycle arrest with features characteristic of senescence. We further show that this senescence-like arrest is dependent on the activity of p53 and Rb, two tumor-suppressor proteins whose activity is frequently de-regulated in NPM-ALK^+^ ALCL [33], [34].

## Results

### NPM-ALK kinase activity induces cell-cycle arrest of primary MEFs with features of senescence

**Figure 1:**
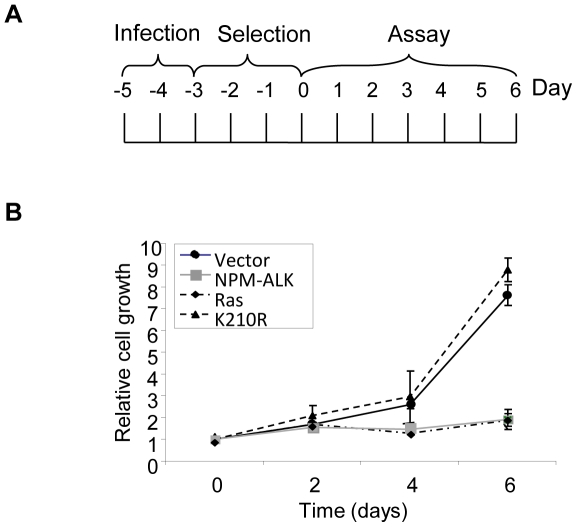
NPM-ALK expression in primary MEFs inhibits cellular proliferation. (A) Experimental design and reference timeframe. Infection refers to exposure of MEFs to retrovirus, and selection refers to enrichment for transduced cells with 2 µg/ml of puromycin. (B) MEFs infected with retrovirus and selected for expression of the indicated genes were assessed for their ability to proliferate over 6 days *via* crystal violet assay. Relative cell growth was determined by comparing crystal violet optical density values obtained at 590 nm at each time point with those obtained at day 0. Each time point was conducted in triplicate in at least 3 separate experiments using MEFs from independent embryo preparations. Error bars are standard deviations of the mean.

Early-passage MEFs were transduced by retrovirus with MSCVpuro vectors encoding NPM-ALK, a kinase-dead mutant of NPM-ALK (K210R), H-Ras V12 or insert-free vector and were selected in puromycin for three days before their use in the assays described below. The timeline of the experiment is depicted in Figure 1A.

**Figure 2:**
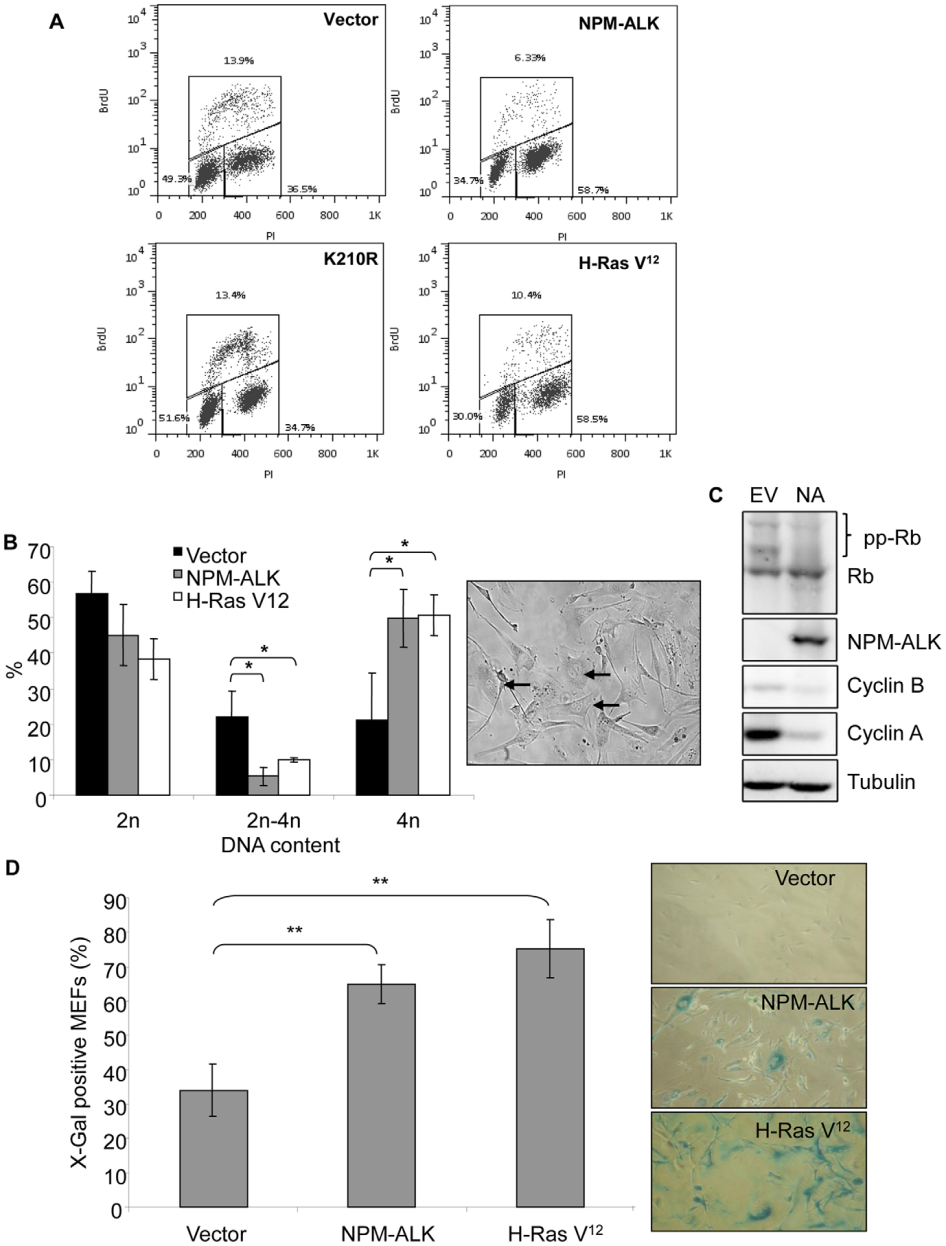
NPM-ALK expression in primary MEFs induces a senescence-like arrest. (A) Representative data of flow cytometric cell-cycle analysis following BrdU incorporation and PI staining. The assay was performed at day 6. Lower-left box identifies cells with 2n DNA content; upper box identifies cells in S phase; Lower-right box identifies cells with 4n DNA content. (B) Graphical representation of cell-cycle analysis (left panel). Data shows the mean of three independent experiments. Error bars are standard deviations of the mean. *denotes significance at p<0.05 using a Student's T-test. Microscopic examination at ×100 magnification showing NPM-ALK cells with 2 nuclei (indicated by arrows) (right panel). (C) Protein lysates prepared from cells transduced with and selected for expression of vectors encoding NPM-ALK (NA) or an empty vector (EV) were examined by Western blot for expression of the indicated proteins with their respective antibodies (pp-Rb indicates phosphorylated Rb). (D) Bright-field images of MEFs (×25 magnification) infected with the indicated retroviruses and assayed for SA-β-Gal activity (indicated by blue coloring) at day 4 (right panel). The bar chart displays quantification of the senescence assay. H-Ras-V12-expressing MEFs were used as a positive control for senescence. At least 200 cells were counted per well (in triplicate in at least 3 separate experiments using MEFs from independent embryo preparations). Error bars are standard deviations of the mean. **denotes significance at p<0.01 using a Student's T-test.

H-Ras V12 is an oncoprotein that induces senescence when expressed in primary MEFs (Figure 1B and [35]). Likewise, it was observed that expression of the oncogenic tyrosine kinase NPM-ALK in MEFs prevented the accumulation of cells as assessed by crystal violet assay (Figure 1B). The amount of cell death was similar between control and NPM-ALK-expressing cells (Figure S1), indicating that the reduced proliferative capacity of the latter was primarily due to cell-cycle arrest rather than cell death. Consistent with this notion, cells remained attached to the plates and DNA synthesis (as measured by BrdU and PI incorporation) was reduced in NPM-ALK-expressing cells to a level comparable to that of H-Ras V12-expressing MEFs (Figure 2A). The cell-cycle arrest was dependent on the kinase activity of NPM-ALK since the kinase-dead K210R mutant showed the same growth kinetics and cell-cycle profile as vector control cells (Figures 1B and 2A).

Cell-cycle analysis of both NPM-ALK- and H-Ras V12-expressing cells revealed a significant decrease in the percentage of cells with 2n–4n DNA content (indicating S-phase) and an accumulation of cells with 4n DNA content (indicating G2/M) (Figure 2A, B). H-Ras V12 expression in primary cells has previously been associated with an arrest of cells in the G1 phase of the cell cycle, therefore to determine whether the accumulation of cells with 4n DNA content was in fact a result of a G1 tetraploid arrest (DNA synthesis in the absence of cytokinesis) cells were subjected to microscopic examination and Western blot analysis for the G2/M marker, cyclin B. At day 4 of the experimental time frame NPM-ALK- and H-Ras V12-expressing cells containing two nuclei were present at high frequency in the population (Figure 2B, right panel and data not shown) and, furthermore, Western blot analysis revealed that Rb was present in NPM-ALK-expressing cells predominantly in its hypophosphorylated (active) form without up-regulation of the G2/M marker cyclin B but with down-regulation of cyclin A (Figure 2C). Together, these data support a predominant tetraploid G1 arrest of NPM-ALK-expressing cells.

Consistent with a senescent arrest NPM-ALK-expressing cells did not resume proliferation after at least a month of culture (data not shown), displayed typical morphological features of senescent cells, becoming enlarged and flattened (Figure 2B, right panel), and a substantial proportion of the cell population stained positively for Senescence-Associated-β-Galactosidase (SA-β-Gal) activity by day 4 of the experimental time-frame (Figure 2D).

### NPM-ALK-induced senescence-like arrest is dependent on p53

**Figure 3:**
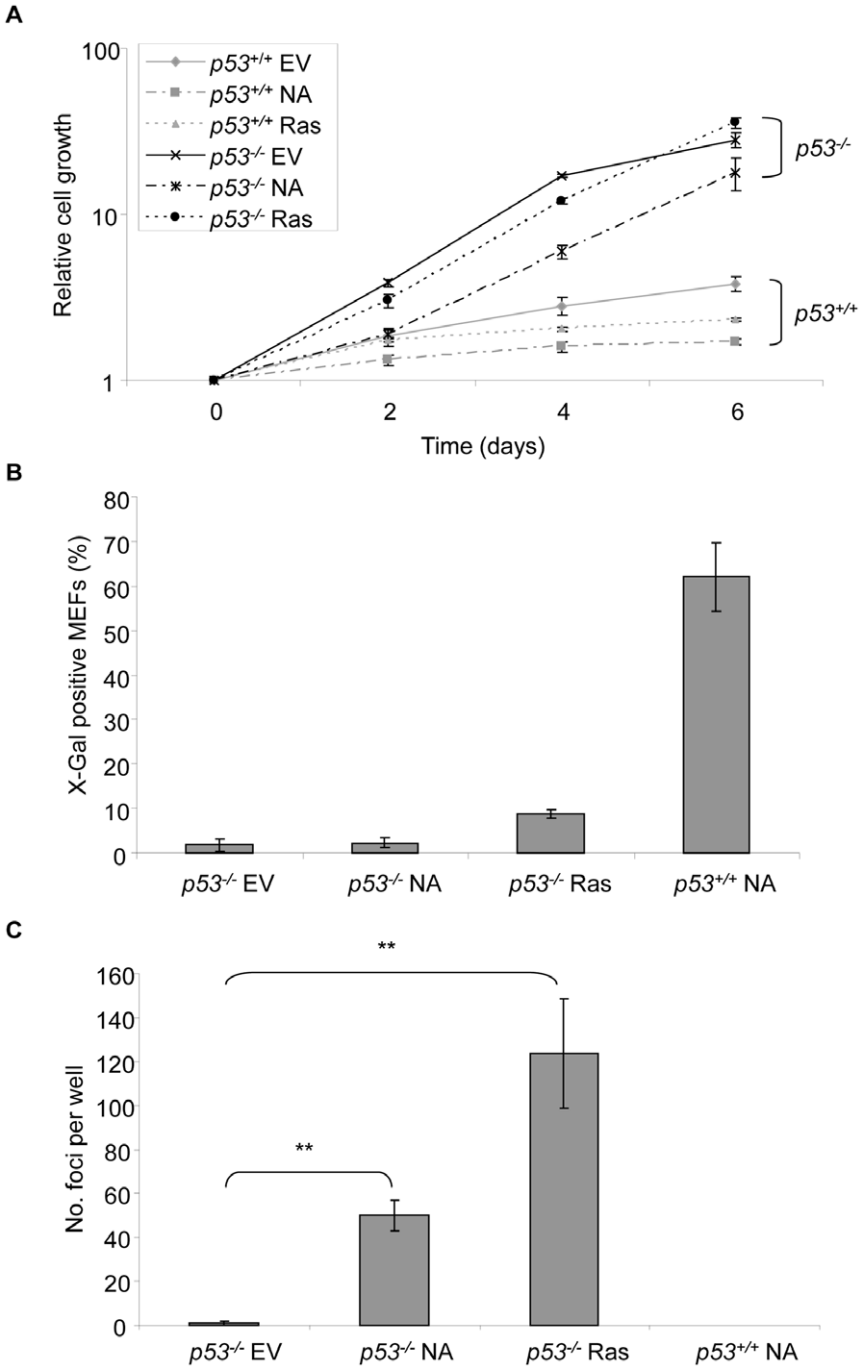
NPM-ALK-induced senescence is p53-dependent. Early-passage *p53^−/−^* MEFs were retrovirally transduced with empty (EV), NPM-ALK- (NA), or H-Ras-V12 (Ras)-containing vectors and selected in puromycin as detailed in Fig. 1A before use in the following experiments: (A) Relative cell growth was determined by comparing crystal violet optical density values obtained at 590 nm at each time point with those obtained at day 0. (B) SA-β-Gal activity was determined at day 4 post-selection. At least 200 cells were counted per well. (C) Anchorage-independent growth in soft agarose was assessed by foci formation at weeks 2–3. Each experiment in (A)–(C) was conducted in triplicate in at least 2 separate experiments using MEFs from independent embryo preparations. Error bars are standard deviations of the mean. **denotes significance at p<0.01 using a Student's T test.

**Figure 4:**
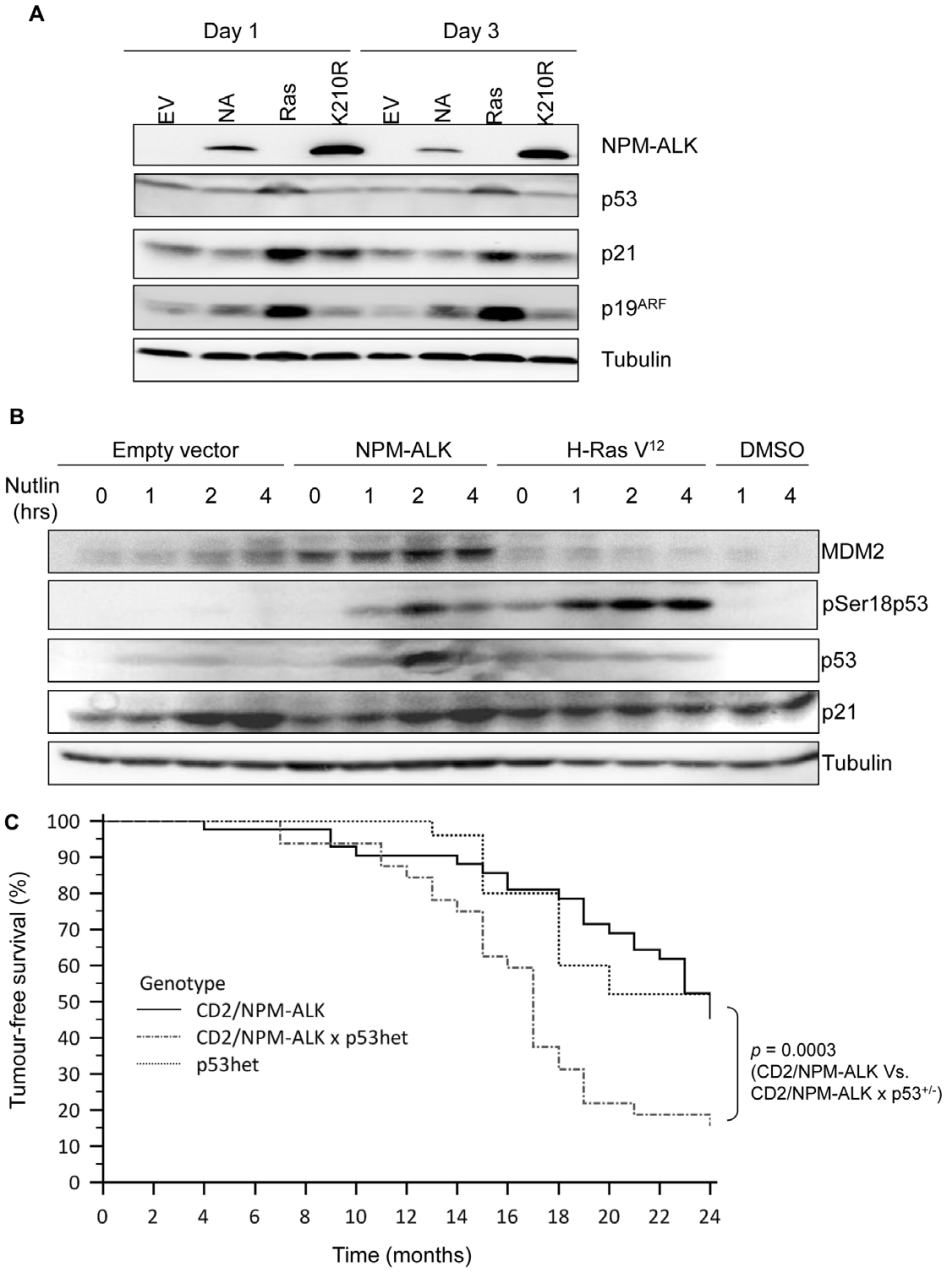
NPM-ALK-induced senescence is p53-dependent but is not due to protein stabilization. (A) Western blot demonstrating lack of p53/p21/p16/p19 up-regulation in response to NPM-ALK expression in MEFs at the indicated time points in comparison to oncogenic H-Ras V12-expressing cells. (B) NPM-ALK-expressing MEFs treated with the MDM2-antagonist Nutlin-3 for the indicated times show stabilization of p53 and transcriptional activity as evidenced by p21 expression, detected by Western blot. (C) CD2/NPM-ALK transgenic mice when back-crossed onto a *p53* heterozygous background displayed a significant increase in tumor development suggesting that p53 delays tumor progression.

H-Ras V12-induced senescence of MEFs requires an intact p19ARF/p53 pathway [35], [36]. To determine if this is likewise the case for NPM-ALK, we expressed NPM-ALK in p53-deficient (p53^−/−^) MEFs and assessed growth and SA-β-Gal staining shortly after retroviral transduction and selection. In the absence of p53, NPM-ALK-induced growth arrest was bypassed (Figure 3A) and the cells no longer stained positively for SA-β-Gal (Figure 3B). In fact, NPM-ALK-expressing p53^−/−^ MEFs lost contact inhibition, were capable of proliferation at low density and could grow without a solid support in soft-agarose, all features consistent with a transformed phenotype (Figure 3C). Despite the involvement of p53 in NPM-ALK-induced senescence, we did not observe stabilization of the protein nor induction of its prototypic, senescence-associated target gene *p21* by Western blot analysis, whereas H-Ras V12 induced both of these proteins at two different time points (days 1 and 3 of the experimental time-frame; Figure 4A), consistent with previous observations [35]. We noted that p19ARF, a protein required to stabilize p53 [37], was induced to only modest levels compared to H-Ras V12 following NPM-ALK expression, which may account for the lack of detectable p53 stabilization in these cells (Figure 4A). Moreover, we have previously reported that NPM-ALK targets p53 for degradation *via* the MDM2 and JNK pathways in transformed human ALCL cell lines [33]. Thus, the lack of p53 stabilization is consistent with our previous findings. Indeed, treatment of NPM-ALK-expressing MEFs with the MDM2 inhibitor, nutlin-3, for 1, 2 and 4 hours was sufficient to stabilize p53 to relatively high levels by 2 hours post-treatment, leading to the induction of p21 and MDM2 (Figure 4B). The return of p53 to basal levels by 4 hours post-treatment may indicate the activation of alternative p53-degradation pathways. Treatment of H-Ras V12-expressing cells in the same manner did not lead to further p53 stabilization nor enhanced p21 induction, presumably indicating that the MDM2-p53 interaction is already maximally disrupted in these cells and also suggesting that NPM-ALK induces expression of p53 whilst simultaneously enabling its degradation (p53 expression levels are much higher in the NPM-ALK expressing cells suggesting that it induces transcription of this protein).

Consistent with a role for p53 in the suppression of cellular transformation by NPM-ALK, transgenic mice in which NPM-ALK expression is driven from the lymphoid-specific CD2 promoter [30] exhibited accelerated lymphoma development and increased tumor incidence when the oncogene was expressed on a *p53* heterozygous genetic background relative to NPM-ALK expression on a *p53* wild-type background (p<0.0005) (Figure 4C). These data are indicative of a p53-imposed delay in tumorigenesis induced by NPM-ALK.

In summary, NPM-ALK induces a senescence-like growth arrest that is dependent on p53 in the absence of further p53 stabilization Alternatively, NPM-ALK induces concomitant transcription and degradation of p53 protein which equilibrate to levels sufficient for senescence induction.

### p16 is dispensable for NPM-ALK-induced senescence

**Figure 5:**
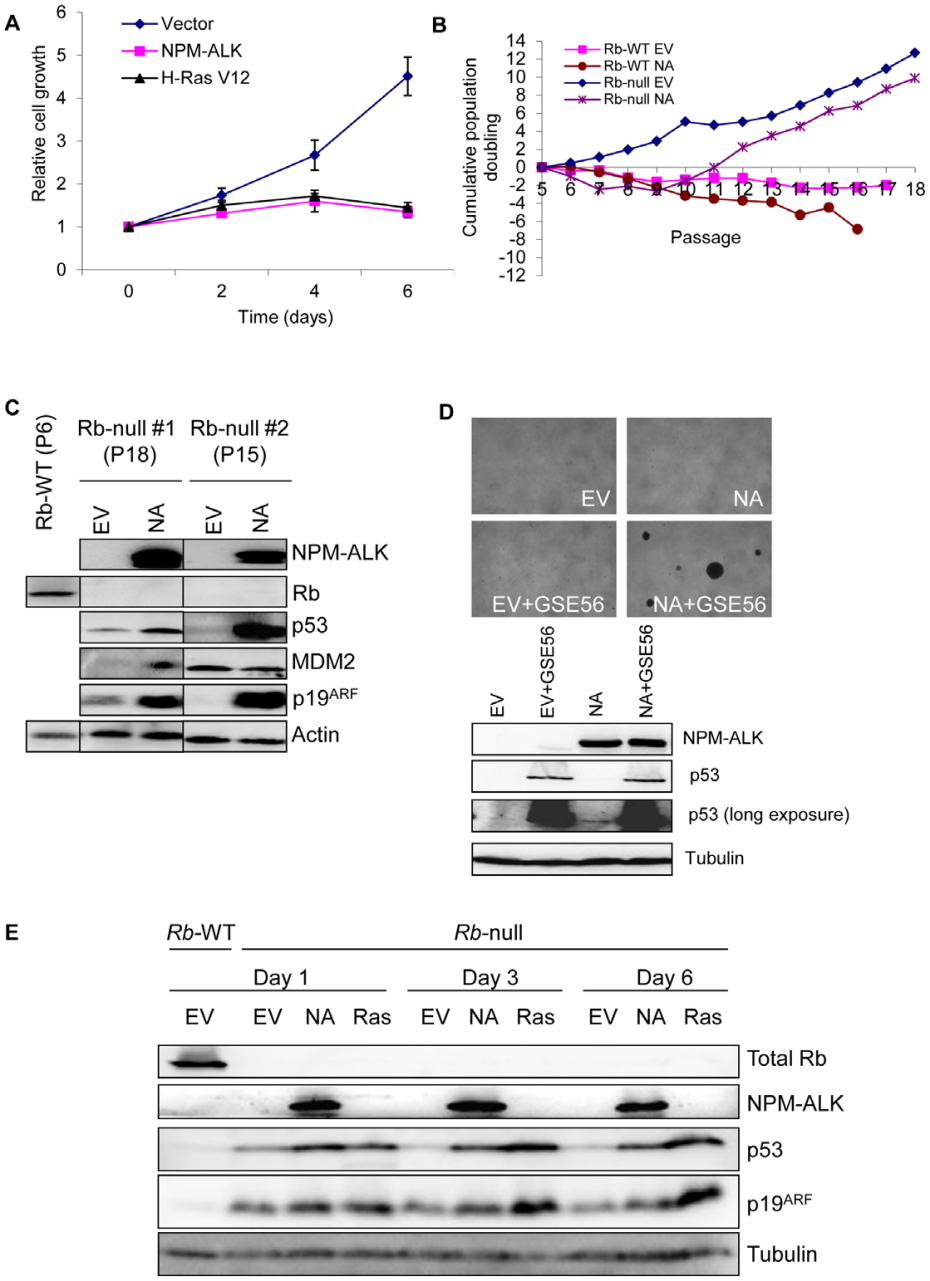
Acute mutation of Rb (but not germline loss of p16) permits escape from NPM-ALK-induced senescence yet the cells remain sensitive to p53-dependent barriers to transformation. (A) Early-passage *p16^−/−^* MEFs were retrovirally transduced with empty (EV), NPM-ALK- (NA), or H-Ras-V12 (Ras)-containing vectors and selected in puromycin as detailed in Fig. 1A before use in a crystal violet assay to assess proliferative capacity. Relative cell growth was determined by comparing crystal violet optical density values obtained at 590 nm at each time point with those obtained at day 0. Each time point was conducted in triplicate in at least 3 separate experiments using MEFs from independent embryo preparations. Error bars are standard deviations of the mean. (B) Floxed cRblox/lox (Rb-null) or control cRblox/lox (Rb-WT) MEFs were transduced by retrovirus with empty (EV) or NPM-ALK- (NA) containing vectors as indicated and used in 3T3 assays to assess long-term proliferative potential. The data shown are representative of two independent *cRb^lox/lox^* MEF populations derived from two different embryos. (C) Western blot analysis showing levels of the indicated proteins in empty-vector and NPM-ALK-expressing conditional Rb-deficient (Rb-null) MEFs at late passage (cells post-senescent at passages 18 and 15 are shown from 2 independent MEF preparation experiments). Horizontal lines on the Western blot indicate repositioning of the gel lanes. (D) Top panel: images of the soft agarose assay (×25 magnification) demonstrating that whilst NPM-ALK-expressing cells acutely mutated for *Rb* have escaped senescence they do not grow in an anchorage-independent manner, and therefore are not transformed (for quantification of data see table 1). Inactivation of p53 by GSE56 expression in these cells results in transformation (lower right box). Bottom panel: Western blot showing NPM-ALK and p53 expression in the different cells used in the soft agarose assay. Experiments were performed twice with two independent MEF populations. (E) p53 is induced following NPM-ALK expression in cells acutely mutated for Rb. Lysates were prepared from MEFs of the indicated genotypes and the levels of Rb, p53, p19ARF and NPM-ALK determined by Western blot. “Rb-null” denotes MEFs in which the *Rb* gene is acutely mutated by addition of Adenovirus expressing Cre-recombinase, and “Rb-WT” denotes MEFs infected with a control Cre-recombinase-deficient Adenovirus.

Another major pathway involved in senescence is the p16/Rb pathway [38]. To address the role of this pathway in NPM-ALK-induced senescence, NPM-ALK was initially expressed in p16-deficient (*p16* ^−/−^) MEFs and the expansion of puromycin-selected cells was assessed by crystal violet assay. We observed that germline loss of *p16* was insufficient to bypass growth arrest induced by both NPM-ALK and H-Ras V12 (Figure 5A).

### Acute mutation of Rb permits bypass of NPM-ALK-induced senescence but is insufficient for transformation

It has previously been shown that following germline mutation of *Rb*, the protein product of which is the downstream target of p16, the Rb-related proteins p107 and p130 can functionally compensate in certain cellular contexts [39], [40]. Therefore to determine a direct role for the Rb pathway in NPM-ALK-induced senescence, NPM-ALK was expressed in conditional *Rb*-knockout (*cRb^lox/lox^*) MEFs in which the *Rb* gene can be irreversibly ablated at will *via* the addition of Adenovirus expressing Cre-recombinase recognizing LoxP-flanked sequences in exon 3 of the *Rb* gene (Figure S2A and [40]). MEFs were infected and selected according to the protocol shown in Figure S2B. Following passage according to a standard 3T3 protocol (see materials and methods) we observed that NPM-ALK-expressing MEFs invariably overcame any initial growth deficit at early passage and exhibited robust proliferation for at least two months (Figure 5B). Paradoxically, Western blot analysis of lysates harvested from 2 independent cell populations cultured under the same conditions at passage 18 and 15 respectively revealed that both p19ARF and p53 levels were highly elevated in NPM-ALK-expressing cells (Figure 5C). Furthermore, p53 was functional in floxed *cRb^lox/lox^* NPM-ALK-expressing cells as revealed by the inability of the cells to adopt a transformed phenotype (loss of contact inhibition and growth in an anchorage-independent manner) unless p53 function was simultaneously disrupted *via* co-infection with a retrovirus encoding the p53 dominant-negative peptide, GSE56 (Figure 5D and Table 1). GSE56 antagonizes p53 function through interaction with its oligomerisation domain, subsequently resulting in the dramatic accumulation of inactive p53 [41] (Figure 5D). Thus, Rb functional loss permits bypass of NPM-ALK-induced senescence but transformation is nevertheless prevented due to p53-imposed restraints. This interplay between the p53 and Rb pathways in senescence bypass and transformation has previously been described for H-Ras V12 and indicates that p53 can act downstream of Rb in the prevention of cellular transformation [40], [42].

**Table 1:** Loss of p53 function is required for anchorage-independent growth of NPM-ALK-expressing conditional Rb-null post-senescent MEFs.

1st infection (P3)(MSCVpuro vector)	2nd infection (P15–18)(MSCVneo vector)	Number of Foci
Empty	Empty	0±0
Empty	GSE56	0±0
NPM-ALK	Empty	0±0
NPM-ALK	GSE56	118±11

P = passage.

### Rb is required for NPM-ALK-mediated p53 degradation

The data presented in Figure 5C further revealed that in the absence of Rb, NPM-ALK activity was sufficient to induce p53 (compare lack of p53 induction in NPM-ALK-expressing cells in wild-type MEFs in Figure 4A and p53 induction in Rb-deficient MEFs in Figure 5C), suggesting that Rb is required for the previously described mechanism of NPM-ALK-mediated p53 degradation [33]. This requirement for Rb was also confirmed in early passage MEFs where p19ARF remained only modestly induced (Figure 5E), and warrants further investigation.

## Discussion

**Figure 6:**
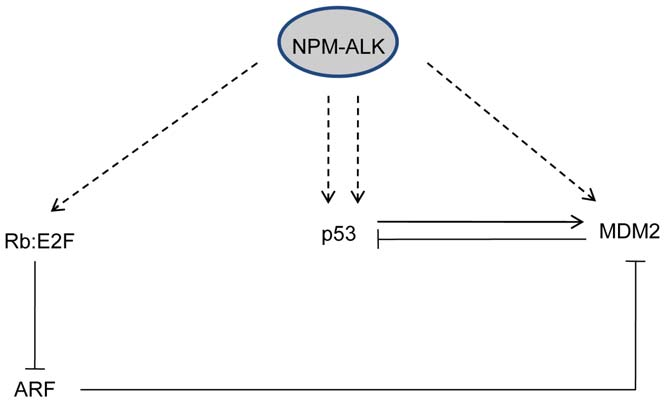
NPM-ALK paradoxically induces transcription and degradation of p53 to generate an equilibrium whereby the dominating residual levels of p53 are sufficient to induce senescence. We propose that NPM-ALK induces transcription of p53 whilst also stabilizing MDM2 and Rb with the effect of attenuating p53 activity. However, equilibrium is reached whereby residual p53 protein is sufficient to set in place a senescence pathway.

Much work has been performed to delineate the mechanism of transformation by oncogenic ALK, with most studies focusing on the activity of NPM-ALK and its role in the genesis of ALCL [18], [19], [21]. Carcinogenesis is generally regarded as a multi-step process requiring an undefined number of mutations and/or epigenetic changes that confer specific growth advantages and/or permit bypass of tumor-suppressive mechanisms [29]. Using primary cell lines we demonstrate here that NPM-ALK triggers a durable proliferative arrest *via* activation of the p53 and Rb pathways with many features characteristic of senescence. These data indicate that loss of p53 or Rb function permits bypass of NPM-ALK-induced cell-cycle arrest, with p53 loss-of-function also leading to transformation. Whilst the *p53* gene is rarely mutated in ALK^+^ ALCL [43] - indeed *p53* mutations are uncommon in a range of hematopoietic cancers [44] – we have previously shown that p53 function is disrupted in ALK^+^ ALCL cell lines through NPM-ALK-mediated p53 degradation in a manner that depends on the activities of MDM2 and JNK [33]. We likewise demonstrate here a dependency on MDM2, as well as Rb, to target p53 for degradation in NPM-ALK-expressing MEFs, albeit at an insufficient level in this case to disrupt p53 function entirely, thus triggering growth arrest and preventing transformation. However, in the context of incipient tumor cells *in vivo*, it is possible that other undefined molecular events co-operate with NPM-ALK to abrogate p53 function (see Figure 6).

The prevention of p53 stabilization and p21 induction following NPM-ALK expression in MEFs may at first glance appear at odds with a role for p53 in preventing NPM-ALK-induced senescence. These data suggest that basal levels of p53 are sufficient to mediate the growth arrest response. Indeed, p53 exists at low basal levels under homeostatic conditions to be functionally activated in response to cellular stress. This can occur as a result of covalent modifications that disrupt the C-terminal negative regulatory domains of p53 tetramers, leading to conformational changes compatible with DNA binding and transactivation of p53-responsive genes [45]. Thus, whilst p53 expression was not detectably increased in response to NPM-ALK activity, there remains the possibility that the basal levels of p53 present are transcriptionally active as a consequence of NPM-ALK-induced post-translational modifications. Furthermore, the repertoire of p53-responsive genes transcribed may be different to H-Ras-V12-expressing cells due to the presence of lower levels of active p53. Thus, p53-transcriptional targets other than p21 may be transcribed to mediate NPM-ALK-induced senescence.

In support of a role for p53 in suppressing NPM-ALK-induced lymphomagenesis, we observed accelerated lymphoma development in CD2/NPM-ALK-transgenic mice expressing a single allele of *p53* (Figure 4C). Assessing the nature of the tumor-suppressor mechanism governed by p53 in this instance of lymphomagenesis is severely hampered by the fact that a dysplastic, pre-tumorigenic state has not been identified in any of the mouse models of ALCL described to date, nor in patients. This is in contrast to the genesis of many solid tumors which can be traced through a series of well-defined histopathological changes in tissue architecture from a benign tumor through to its malignant conversion. Although it remains technically challenging to identify a putative precursor cancer lesion that may occur anywhere in the lympho-hematopoietic compartment, senescence has been identified as an anti-tumor mechanism in mouse models of lymphoma development driven by oncogenic Myc or N-Ras [46], [47]. Thus, identifying a role for senescence in the suppression of NPM-ALK-driven lymphomagenesis may yet be possible.

Germline loss of *p16* is not sufficient to bypass NPM-ALK-induced senescence; however, given the occurrence of functional compensation by related proteins in biological systems we cannot exclude the possibility that sporadic loss of p16 during tumor evolution is sufficient to circumvent senescence. Nevertheless, mutations or epigenetic modifications affecting the *p16* gene are not common in ALCL [48]. In contrast, the *Rb* gene is frequently mutated or the encoded protein hyperphosphorylated (and therefore inactive) in ALK^+^ ALCL [34]. Consistent with this observation, we found that acute mutation of *Rb*, which mimics sporadic mutation and limits functional compensation by the Rb-related proteins p107 and p130, circumvents NPM-ALK-induced senescence. We propose that loss of Rb function in the evolution of NPM-ALK^+^ ALCL may be sufficient to counteract NPM-ALK-induced activation of cell-cycle checkpoints, permitting unabated and unchecked progress through the cell cycle. In this manner the likelihood of acquiring additional mutations necessary for full-blown or more aggressive malignancy, such as those affecting p53 function, may be enhanced.

Whilst MEFs do not represent the physiological target of NPM-ALK in lymphomagenesis (indeed, the ALCL cell of origin remains unknown), this work has highlighted the potential existence of tumor-suppressive barriers during the development of ALCL. Moreover, given that the transforming activity of NPM-ALK seems to be confined to the ALK portion of the protein through activation of the kinase domain [17], this work also raises the possibility that p53 and/or Rb activation followed by cell-cycle arrest may constitute a more wide-spread phenomenon in the genesis of other cancers in which ALK is aberrantly activated. It furthermore provides a rationale to explore senescence induction as a potential therapeutic strategy in ALK-driven cancers.

## Materials and Methods

### Cells

MEFs (derived from E12.5-E13.5 embryos) and Phoenix cells were cultured in Dulbecco's Modified Eagles Medium (DMEM) containing 4500 mg/L D-Glucose and 110 mg/L Sodium Pyruvate (Sigma-Aldrich), 10% fetal calf serum (FCS; Invitrogen, Paisley, UK), 2 mM L-glutamine (Invitrogen) and 1% Penicillin and Streptomycin (Gibco, Scotland, UK). MEFs were utilized at passage 1–2. *cRb^lox/lox^* and *p16^−/−^* MEFs were kindly provided by Prof. Julien Sage (Stanford University, CA) and Prof. Pier Giuseppe Pelicci (European Institute of Oncology, Milan, Italy) respectively.

### Antibodies

Cyclin B1 (V152) and MDM2 (ser166) antibodies were purchased from Cell Signaling Technologies (Danvers, MA); Cyclin A, α-tubulin and β-actin from Sigma-Aldrich (Poole, UK); p19 (5-C3-1), p21 (F-5) and MDM2 (SMP14) from Santa Cruz (Heidelberg, Germany); p53 (CM5) from Novocastra (Newcastle-Upon-Tyne, UK); Rb (G3-245) from BD Biosciences (Oxford, UK); ALK from Zymed (San Francisco).

### Inhibitors

MEFs were treated with 10 µM of a racemic mixture of the MDM2 antagonists, Nutlin-3a and Nutlin-3b (Calbiochem), for the indicated periods at 37°C/5% CO2.

### Plasmids

Human NPM-ALK cDNA was cloned into the Murine Stem Cell Virus (MSCV) puro vector. MSCVpuroK210R was generated *via* site-directed mutagenesis of MSCVpuroNPM-ALK using a site-directed mutagenesis kit (Stratagene, Cheshire, UK), following the manufacturer's protocol. Primer pairs K210R_Forward 5′-CCCTGCAAGTGGCTGTGAGGACGCTGCCTGAAGTGTTGC-3′ and K210R_Reverse 5′-GCAACACTTCAGGCAGCGTCCTCACAGCCACTTGCAGGG-3′ were used. GSE56 was cut from pL56-RAS (a gift from Dr Andrei Gudkov, Roswell Park Cancer Institute, NY) and ligated into MSCVneo. pBabepuroH-Ras V12 was a gift from Dr Masashi Narita (Cambridge Research Institute, UK).

### Retroviral and adenoviral infection of MEFs

Phoenix cells were transfected with the indicated plasmid vector(s) and a murine packaging “helper” construct, ϕ, using Fugene transfection reagent (Roche, West Sussex, UK) following the manufacturer's protocol. At 48 hours post-transfection, the virus-containing media was harvested from the phoenix cells, filtered through a 0.45 µm filter, supplemented with 4 µg/ml polybrene (Sigma-Aldrich) and used to infect 8×10^5^–1.2×10^6^ MEFs in a 10 cm dish at 32°C/5% CO_2_. MEFs were selected in 2 µg/ml puromycin (Sigma-Aldrich) 48 hours post-infection for 2–3 days or 300 µg/ml geneticin (Gibco) for 7–10 days.

Adenovirus expressing recombinant Cre-recombinase from a CMV promoter (AdCre) and insert-free adenovirus control (AdEmpty) were purchased from the Gene Transfer Vector Core, University of Iowa, IA. Virus (5 µl) was added to 8×10^5^–1.2×10^6^ Cre-responsive MEFs seeded into a 10 cm tissue-culture dish in 10 ml complete media, followed by incubation at 37°C/5% CO_2_ for 72 hours. A second round of infection was performed 16–24 hours later.

### Western blot analysis

Western blot was performed as previously described (32), using RIPA buffer (50 mM Tris-HCl, pH 7.4; 1% NP-40; 0.25% Na-deoxycholate; 150 mM NaCl; 0.1% Sodium dodecyl sulphate (SDS); 1 mM Ethylenediaminetetraacetic acid (EDTA); 1 mM Phenylmethylsulphonyl fluoride (PMSF); 2 mM Na_3_VO_4_; 20 mM NaF; and protease inhibitor cocktail tablet (Roche)) to lyse the cells.

### Proliferation assays

Crystal violet assays were performed by seeding MEFs into 12-well plates at a density of 2.5×10^4^ cells/well (in triplicate) which were fixed following incubation for the indicated times with 1% glutaraldehyde (Sigma-Aldrich) in PBS. Cells were washed with PBS, stained with 0.1% crystal violet (Sigma-Aldrich) for 30 minutes and crystal violet extracted with 10% (v/v) acetic acid (Sigma-Aldrich) in distilled water. The OD at 590 nm was measured with a 1420 Multilabel VICTOR3 plate reader (PerkinElmer, MA).

A 3T3 assay was performed according to the protocol of Todaro and Green [49]. In brief, cells were passaged every 3 days at 1.2×10^6^ cells in 10 cm plates even in the absence of growth between passages. If the cell number declined, cells were maintained at an equivalent density by seeding at 3×10^5^ in a 5 cm plate or at 1×10^5^ in a 3.5 cm plate. Population doubling was calculated using the equation:
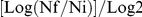
Nf is the final cell number counted after 3 days, and Ni is the initial cell number seeded.

### Apoptosis

Cells were washed twice in cold PBS followed by resuspension in binding buffer (0.01 M 4-(2-hydroxyethyl)-1-piperazineethanesulfonic acid [HEPES], 0.14 M NaCl and 2.5 mM CaCl_2_). The cells were incubated with 5 µl APC-conjugated Annexin V antibody (BD Biosciences) and 5 µg/ml PI at room temperature in the dark for 15 minutes before analysis on a FACSCalibur cytometer (BD Biosciences). Data were analyzed using FlowJo software (TreeStar, Ashland, OR).

### Cell-cycle analysis

1×10^6^ cells in 10 cm dishes were incubated with 10 µM BrdU for 4 hours followed by fixation with 70% ice-cold ethanol/30% PBS. Cells were washed twice in PBS, resuspended in 500 µl 2 M HCl and incubated for 20 minutes at RT, then rinsed twice in wash buffer (0.5% bovine serum albumin [BSA, Sigma-Aldrich] in PBS). A 0.1 M solution of sodium borate (500 µl) (Sigma-Aldrich) was then added followed by incubation at room temperature for 2 minutes, followed by 2 rinses in wash buffer. Cells were resuspended in 20 µl fluorescein isothiocyanate (FITC)-conjugated mouse anti-BrdU monoclonal antibody (BD Biosciences) and incubated for 20 minutes in the dark. Cells were then washed twice and resuspended in 500 µl PBS, 0.1 mg/ml RNase A (Sigma-Aldrich), 0.4 mg/ml PI (Sigma-Aldrich) and incubated at 37°C for 30 minutes. Cells were then passed several times through a 25-gauge needle and analyzed on a FACSCalibur cytometer (BD Biosciences). A minimum of 20,000 events were collected per sample and the data analyzed using FlowJo software.

### SA-β-Gal assay

2.5×10^4^ cells/well of a 12-well plate (in triplicate) were fixed with 0.5% glutaraldehyde for 15 minutes at RT. Cells were washed twice in 1 M MgCl_2_ (Sigma-Aldrich) in PBS, pH5.5 and incubated in 1 ml of X-Gal staining solution (1 mg/ml X-gal [Sigma-Aldrich], 0.12 mM K_3_Fe[CN]_6_, 0.12 mM K_4_Fe[CN]_6_, 1 mM MgCl_2_ in PBS at pH5.5) overnight at 37°C. The cells were washed 3 times in dH_2_O and images captured with a Leica D-LUX 3 camera mounted onto a Labovert microscope at ×100 magnification. The percentage of cells staining positive (blue color) for β-galactosidase activity was determined by counting the cells by eye at ×40 magnification. At least 200 cells were counted per well.

### Mice

CD2/NPM-ALK transgenic mice were produced as detailed in [30]. The *p53^+/−^* mice were kindly provided by Prof. Terry Rabbitts, Leeds Institute for Molecular Medicine, UK and were bred on a C57/BL6 background. All mice were housed under spf conditions at the University of Cambridge in accordance with UK Home Office guidelines. The genotypes of the mice were determined as described in [30] and [50], except that the primer directed against the neo cassette in the latter case had the sequence 5′-TCC TCG TGC TTT ACG GTA TC-3′. The Kaplan-Meier curve and log-rank test were generated in MedCalc.

### Statistical analysis

Data were analyzed using a Student's two-tailed T test (assuming equal variance). Data were considered significant when p values were less than .05.

## Supporting Information

Figure S1 **NPM-ALK does not induce apoptosis.** Annexin V versus PI staining of day 4 primary MEFs followed by FACS analysis demonstrates that NPM-ALK does not induce apoptosis relative to vector-control MEFs. Results are representative of 3 independent experiments. At least 10,000 events were collected for each genotype.
(TIF)

Figure S2 **Ablation of Rb in NPM-ALK-expressing MEFs.** (A) Western Blot analysis of Rb and NPM-ALK expression in early passage *cRb^lox/lox^* MEFs infected with Cre-recombinase-expressing (+) or Cre-recombinase-deficient Adenovirus (−) for 72 hours, followed by infection with vector-only or *NPM-ALK*-encoding retrovirus for 48 hours. (B) Experimental design and time-frame for assays using *cRb^lox/lox^* MEFs.
(TIF)
