# Supplementary figures and images for: Aberrant Anaplastic Lymphoma Kinase Activity Induces a p53 and Rb-Dependent Senescence-Like Arrest in the Absence of Detectable p53 Stabilization
Source: PLoS One. 2011 Mar 14;6(3):e17854. doi: 10.1371/journal.pone.0017854 (PMC3056788; doi:10.1371/journal.pone.0017854)

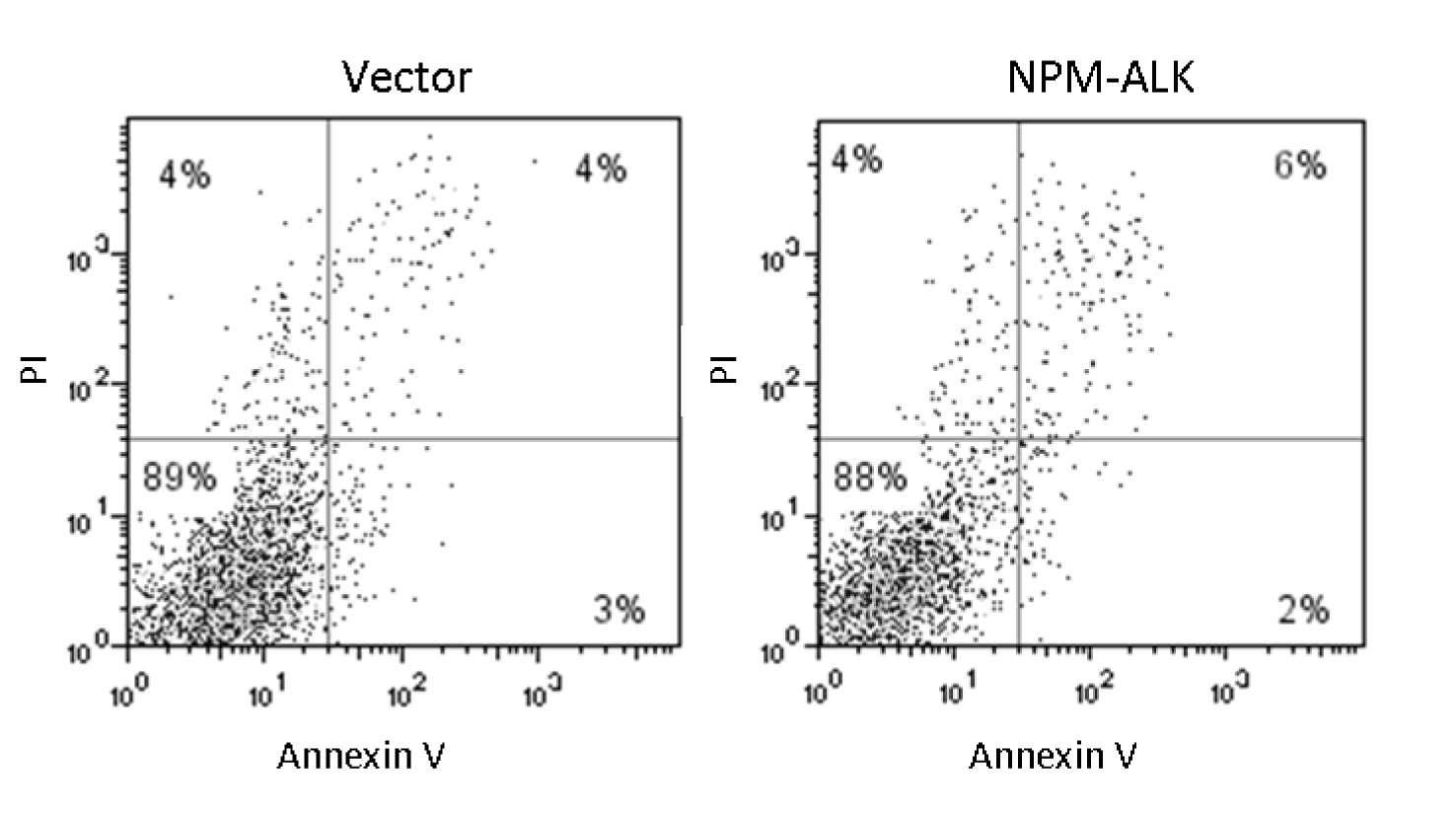

Supplement: Figure S1 — NPM-ALK does not induce apoptosis. Annexin V versus PI staining of day 4 primary MEFs followed by FACS analysis demonstrates that NPM-ALK does not induce apoptosis relative to vector-control MEFs. Results are representative of 3 independent experiments. At least 10,000 events were collected for each genotype. (TIF) [file pone.0017854.s001.tif]

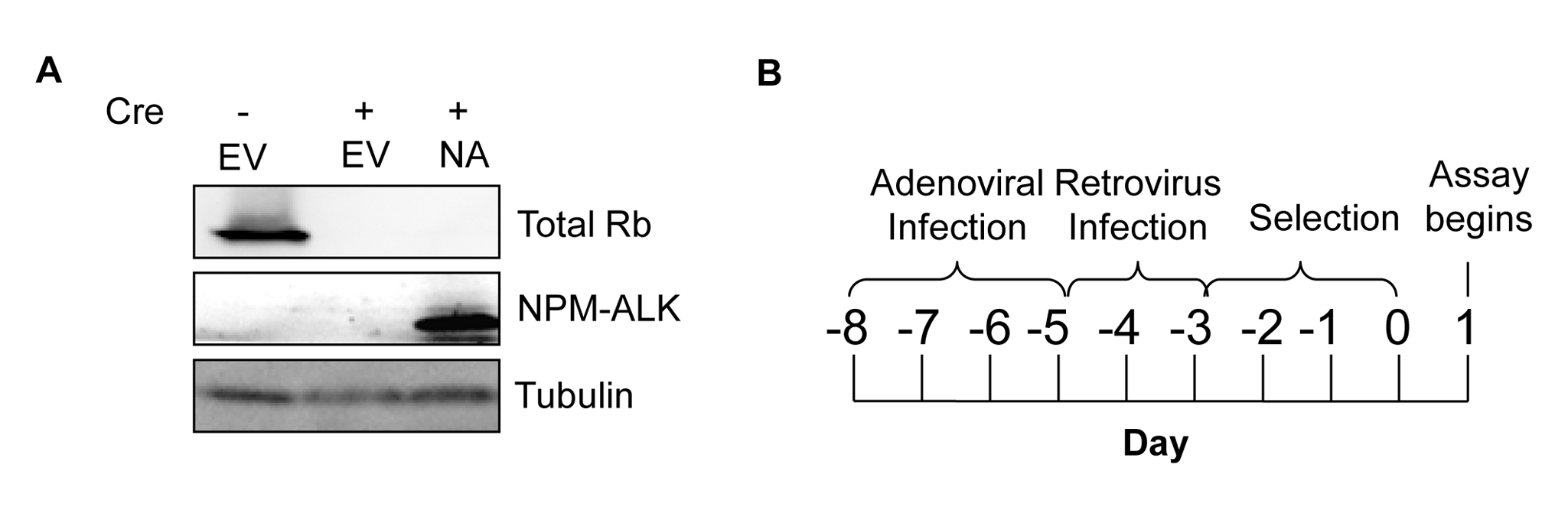

Supplement: Figure S2 — Ablation of Rb in NPM-ALK-expressing MEFs. (A) Western Blot analysis of Rb and NPM-ALK expression in early passage cRblox/lox MEFs infected with Cre-recombinase-expressing (+) or Cre-recombinase-deficient Adenovirus (−) for 72 hours, followed by infection with vector-only or NPM-ALK-encoding retrovirus for 48 hours. (B) Experimental design and time-frame for assays using cRblox/lox MEFs. (TIF) [file pone.0017854.s002.tif]
